# Enhancing Dynamic Balance and Postural Stability in Stroke Patients: The Impact of Immersive Virtual Reality Training

**DOI:** 10.7759/cureus.66299

**Published:** 2024-08-06

**Authors:** Bright Alwin Victor, Arunachalam R, Sheela Angel I, Gnanesh Kumar B

**Affiliations:** 1 Neuro Physiotherapy, Madhav University, Pindwara, IND; 2 Neuro Physiotherapy, Vels Institute of Science, Technology & Advanced Studies, Chennai, IND

**Keywords:** fall risk, postural stability, dynamic balance, stroke, virtual reality

## Abstract

Introduction

Stroke is a major neurological event resulting from reduced or blocked blood flow to the brain, leading to significant morbidity. Immediate medical attention is essential to minimize brain damage and improve outcomes since it leads to many clinical deficits like locomotor impairment, instability in postural control, tonic alterations of the affected musculature, and an array of neurological dysfunctions if left unnoticed. Immersive virtual reality (VR) has emerged as a novel therapeutic tool in stroke rehabilitation, offering engaging and realistic environments for therapy. This study aims to evaluate the effectiveness of immersive VR training combined with functional gait exercises in improving dynamic balance and postural stability in stroke patients, compared to VR training alone.

Methods

This comparative study included 30 subjects from Madha Medical College and Hospital, Chennai, Tamil Nadu, India, divided into two groups. Group A (n=15) received immersive VR combined with functional gait exercises, while Group B (n=15) received immersive VR alone. Subjects were aged 40-60 years with stable blood pressure and a stroke duration of two weeks to six months. The study spanned 12 weeks, with 30-minute sessions on alternate days. Dynamic balance and postural stability were assessed using the Functional Gait Assessment (FGA) and Falls Efficacy Scale (FES). Pre-test and post-test scores were evaluated using parametric tests.

Results

Post-test mean values showed significant improvements in both groups. Group A demonstrated greater effectiveness, with lower FES scores (mean 36.66 ± 11.12) than Group B (mean 46.66 ± 9.75). FGA scores were higher in Group A (mean 28.00 ± 0.925) compared to Group B (mean 26.06 ± 1.66). Significant differences were observed in pre-test and post-test values within each group, supporting the hypothesis that combined VR and gait exercises offer superior rehabilitation outcomes.

Conclusions

Immersive VR combined with functional gait exercises significantly improves dynamic balance and postural stability in stroke patients compared to VR alone. This integrated approach can enhance motor function recovery, increase independence, and improve the quality of life. VR's capability to simulate real-life activities and provide immediate feedback allows for personalized rehabilitation programs. Further research is required to validate these findings and optimize VR-based rehabilitation protocols.

## Introduction

When blood flow to a portion of the brain is significantly reduced or blocked, brain tissue is deprived of oxygen and nutrients, resulting in a stroke [1]. This deprivation leads to brain tissue death and major neurological consequences. A stroke can cause sudden weakness or numbness in one or both arms, legs, or the face, particularly on one side of the body. It can also lead to sudden confusion, difficulty speaking or understanding speech, vision problems in one or both eyes, dizziness, trouble walking, loss of balance or coordination, and a severe headache with no apparent cause. Immediate medical attention is vital for anyone experiencing stroke symptoms, as early treatment can minimize brain damage and improve outcomes.

Neuropsychiatric ailments are a prevalent and especially disruptive negative consequence of stroke. It may be extremely challenging to determine which symptom is the sole physiological consequence of the cerebrovascular accident; these medical conditions emanate from an entire spectrum of psychological distress corresponding to the incident, its subsequent effects, and cognitive impairment brought on by the lesion [2]. The most prevalent of these symptoms comprise anxiousness, depressive disorders, weariness, apathy, emotionalism, and frustration along with other systematic dysfunctions like ambulatory impairment, tonal abnormalities, speech disturbances, equilibrium dysfunctions, and hindrance in overall activities of daily living. Posttraumatic stress disorder associated with the stroke event has also been recognized more widely as a major phenomenon.

The burden of stroke encompasses the significant impact on individuals, families, communities, and healthcare systems. This burden can be understood in terms of health impact, mortality, economic impact, and social impact [3]. Immersive virtual reality (VR) is a type of VR experience that fully engages the user's senses, creating a profound sense of presence and immersion within a digital environment. In immersive VR, users feel as though they are physically present in the virtual world, and their interactions with the environment are highly realistic and natural [4,5].

Dynamic balance refers to the ability to maintain stability and control while in motion or performing dynamic activities involving coordination of the musculoskeletal system, sensory systems (such as proprioception and vestibular function), and the nervous system [6,7]. Dynamic balance training in VR offers an innovative approach to improving balance and stability in a controlled and engaging environment. VR technology can simulate various dynamic and challenging scenarios requiring users to maintain balance, react to perturbations, and perform dynamic movements while immersed in a virtual world [8,9].

Postural stability refers to the ability to maintain an upright posture and control the body's position against the force of gravity, essential for maintaining balance and equilibrium while standing, sitting, or moving [10]. VR has become popular in modern rehabilitation for stroke therapy and has been quickly adopted in healthcare environments. Immediately following a cerebrovascular accident, many people struggle with ambulation, cognition, and sensory functions [11]. This often presents difficulties in performing regular activities like walking, standing, and climbing stairs. Individuals may undergo therapy using VR and interactive video games [12]. This therapy includes computer-based programs that simulate real-world objects and events. Unlike traditional rehabilitation techniques, VR and interactive gaming offer unique benefits by enabling individuals to engage in daily activities [13]. Immersive VR in stroke rehabilitation uses interactive computer-generated environments that simulate real-life activities designed to engage stroke patients in therapeutic tasks to improve dynamic balance and postural stability. These immersive digital environments and interactive technologies challenge and enhance stroke patients' ability to maintain postural stability through exercise and activities [14].

VR provides an engaging, adaptable, and safe environment where patients can practice dynamic balance and postural control tasks. The immersive nature of VR can enhance the intensity and variability of exercises, which are key factors in neuroplasticity and functional recovery. This technological approach allows for the simulation of real-life scenarios in a controlled setting, which could potentially lead to more effective rehabilitation outcomes. Despite being a booming technique, there exists a limited amount of high-quality evidence specifically evaluating the impact of immersive VR training on dynamic balance and postural stability in stroke patients. Most studies have focused on general motor recovery or used non-immersive VR tools. This gap highlights the need for rigorous investigation into how immersive VR can uniquely contribute to balance and postural stability improvements.

Since the current study aims to augment postural stability and dynamic balance, which act as two primary key parameters contributing to locomotion, functional gait exercises were recruited as an add-on with immersive VR as these exercises are easily reproducible and doable for the subjects to enhance their locomotor abilities. Comparing the outcomes of patients receiving this combined therapy with those undergoing immersive VR training alone, we sought to determine whether integrating functional gait exercises with VR can enhance rehabilitation outcomes, thereby providing a more effective approach to stroke recovery. This study also aims to explore the potential of VR technology to offer personalized and engaging rehabilitation experiences that can be implemented both in clinical settings and remotely.

## Materials and methods

This study was a single-blinded, comparative study employing an experimental design, for which 30 subjects were selected from Madha Medical College and Hospital, Chennai, Tamil Nadu, India. All participants were informed about the study procedures and provided written informed consent. The study was approved by the Madha College of Physiotherapy Institutional Review Board (approval number: ABV-18/P-MCP/PHYSIO/IRB/2021-2022).

Selection criteria

The study included both sexes aged 40-60 years, with stable blood pressure, systolic pressure of 110-130 mmHg, diastolic pressure within the range of 70-90 mmHg throughout the study duration, and ischemic stroke duration of two weeks to six months. The exclusion criteria were subjects with lower limb joint contractures, cardiovascular issues, visual impairments, cognitive insufficiency, or ataxia. The 30 subjects chosen based on the inclusion criteria were divided into two groups utilizing the fish bowl sampling method with Group A (n=15) receiving immersive VR alongside functional gait exercises and Group B (n=15) receiving immersive VR alone.

The study lasted 12 weeks, with sessions occurring on alternate days. Each session lasted 30 minutes. The independent variable was VR, while the dependent variables, dynamic balance, and postural stability were assessed using the Functional Gait Assessment (FGA) and Falls Efficacy Scale (FES). Participants in both groups provided written consent before the study began, and we measured pre-test scores. We re-evaluated participants using the FGA and FES after the intervention.

Intervention

*Group A: immersive VR Training and Functional Gait Exercis*e

Static hip march: The patient stood in front of a screen in a precise circle. The therapist instructed the patient to step on virtual objects that matched the color displayed on the screen. Red and yellow virtual objects appeared, and the patient had to step on the virtual object with the matching colored foot until it disappeared while keeping the other foot on the colored footstep. Each session lasted five minutes.

Lower limb abduction: The patient stood in front of a screen in a precise circle. The therapist instructed the patient to place their lower limb on a vertical virtual object presented laterally as an obstacle. The patient had to step on the virtual object with the lower limb until it disappeared, completing the exercise. Each session lasted five minutes.

Standing foot tapping: The patient stood in front of a screen in a precise circle. The therapist instructed the patient to move and hit virtual objects on the floor with their feet. The patient saw shapes marked with numbers on the floor in the virtual room and had to touch them in the correct order. Each session lasted five minutes.

Standing obstacle crossing: The patient stood in front of a screen in a precise circle. The therapist instructed the patient to lift one leg at a time in an alternating pattern to move the virtual image forward while avoiding obstacles. The virtual image traveled down a virtual street, and the patient had to move their legs up and down to advance on the screen. Each session lasted five minutes.

Group B: Immersive VR training

Static hip march and standing foot tapping, as explained for Group A, was done.

Outcome measures

FGA

The FGA is a standardized clinical tool designed to evaluate gait and balance in individuals, particularly those at risk for falls. It assesses how well a person can perform various gait-related tasks that simulate real-life challenges, such as walking over uneven surfaces, changing speeds, and walking while performing additional tasks. It includes tasks like walking over obstacles, walking with head turns, walking backward, and walking while dual-tasking. The FGA consists of 10 items, each scored on a scale from 0 to 3, with higher scores indicating better performance, and the total score ranges from 0 to 30, with higher scores reflecting better functional gait and balance abilities. Lower scores suggest greater balance deficits and higher fall risk [15].

FES

The FES includes questions about various activities that could be challenging for individuals at risk of falling, such as walking, climbing stairs, and getting in and out of bed. FES has demonstrated high internal consistency typically with Cronbach’s alpha values above 0.70 and test-retest reliability, indicating that it consistently measures the same construct over time. It is widely used in research and clinical practice to assess individuals' confidence in their ability to avoid falls, with both validity and reliability being important considerations. FES has demonstrated high internal consistency typically with Cronbach’s alpha values above 0.70 and test-retest reliability, indicating that it consistently measures the same construct over time [16].

Statistical analysis

We employed descriptive and inferential statistics to tabulate and analyze the gathered data. We used IBM SPSS Statistics for Windows, Version 24.0 (Released 2016; IBM Corp., Armonk, New York, United States) for all evaluations. We set a 95% confidence interval for each analysis and applied a significance level of p < 0.05. The Shapiro-Wilk test verified the data's normality. The data in this study were found to be normally distributed on the dependent values at (P > 0.05). Therefore, we used parametric tests. The independent t-test determined the statistical difference between the groups, while the paired t-test assessed the statistical disparity within the groups.

## Results

The post-test mean values in both groups decreased substantially, as indicated by the comparative analysis of Group A and Group B's FES scores. There was no significant difference in pre-test values between Group A and Group B (P > 0.05). However, there was a statistically significant difference in post-test values between Group A and Group B (P ≤ 0.05) (Table 1). Group A, which included dynamic balance in static, lower limb abduction, standing foot tapping, and standing obstacle crossing, showed a mean value of 36.66 ± 11.12, which was more effective than Group B, which included dynamic balance in static and gait walking in straight (mean value of 46.66 ± 9.75, significance 0.001 at P ≤ 0.05). Thus, the null hypothesis was rejected.

**Table 1:** Comparison of pre-test and post-test FES scores in Group A and Group B This table shows that there is no significant difference in pre-test values between Group A and Group B at P > 0.05, and statistically significant difference in post-test values between Group A and Group B at P ≤ 0.05. * P ≤ 0.05; ** P ≤ 0.01, depicting the significance level of p-value DF: degree of freedom; FES: Falls Efficacy Scale

Test	Group A		Group B		T-Test	DF	P-Value
	Mean	SD	Mean	SD			
Pre-test	80.66	8.83	81.33	9.15	-.203	28	.841*
Post-test	36.66	11.12	46.66	9.75	-2.61	28	.014**

The follow-up examination mean values in both groups were substantially higher, according to the comparison of Group A and Group B's mean values on the FGA score. There was a statistically significant difference between the pre-test and post-test values within Group A and Group B (P ≤ 0.05) (Table 2).

**Table 2:** Comparison of pre-test and post-test FGA scores in Group A and Group B This table shows that there is no significant difference in pre-test values between Group A and Group B, and statistically significant difference in post-test values between Group A and Group B at P > 0.05. * P ≤ 0.05;  ** P ≤ 0.01, depicting the significance level of p-value DF: degree of freedom; FGA: Functional Gait Assessment

Test	Group A		Group B		T-Test	DF	P-Value
	Mean	SD	Mean	SD			
Pre-test	16.26	1.62	16.20	1.82	.106	28	.916*
Post-test	28.00	.925	26.06	1.66	3.92	28	.001**

Group A, with dynamic balance in static, lower limb abduction, standing foot tapping, and standing obstacle crossing, showed a higher mean value of 28.00 ± 0.925, which was more effective than Group B, with dynamic balance in static and standing foot tapping (mean value of 26.06 ± 1.66, significance 0.001 at P ≤ 0.05). Hence, the null hypothesis was rejected. Comparing the pre-test and post-test values within Group A and Group B on the FES and FGA scores showed a significant difference in the mean values (P ≤ 0.05) (Tables 3, 4).

**Table 3:** Comparison of FES scores in Group A and Group B pre-test and post-test There is a statistically significant difference between the pre-test and post-test values within Group A and Group B at P ≤ 0.05. ** P ≤ 0.01, depicting the significance level of p-value FES: Falls Efficacy Scale

Group	Pre-Test		Post-Test		T-Test	P-Value
	Mean	SD	Mean	SD		
Group A	80.66	8.83	36.66	11.12	11.34	.000**
Group B	81.33	9.15	46.66	9.75	20.98	.000**

**Table 4:** Comparison of FGA scores in Group A and Group B pre-test and post-test There is a statistically significant difference between the pre-test and post-test values within Group A and Group B at P ≤ 0.05. **P ≤ 0.01 depicting the significance level of p-value FGA: Functional Gait Assessment

Group	Pre-Test		Post-Test		T-Test	P-Value
	Mean	SD	Mean	SD		
Group A	16.26	1.62	28.00	.925	-21.43	.000**
Group B	16.20	1.82	26.06	1.66	-21.62	.000**

## Discussion

Immersive VR represents a cutting-edge technology with numerous potential applications in both current and future rehabilitation practices. Employing virtual games as therapeutic tools, immersive VR-assisted rehabilitation can motivate patients to participate actively in their recovery. The findings of this investigation revealed significant improvements being observed in both FES and FGA scores following the interventions. Although pre-test values for the FES showed no significant difference between Group A and Group B (P > 0.05), post-test values revealed a notable distinction (P ≤ 0.05). Group A, which engaged in immersive VR training incorporating dynamic balance exercises, demonstrated a more effective improvement with a mean FES score of 36.66 ± 11.12, compared to Group B, which underwent training via VR alone with a mean score of 46.66 ± 9.75 (P = 0.001). Additionally, the follow-up FGA scores further supported these findings, with Group A showing a mean score of 28.00 ± 0.925, significantly higher than Group B's 26.06 ± 1.66 (P = 0.001). These results underscore the superior impact of immersive VR training in enhancing both dynamic balance and postural stability in stroke patients, leading to the rejection of the null hypothesis and confirming the effectiveness of the VR intervention.

According to a study by Karasu et al., stroke patients could benefit from Wii Fit (Nintendo Co., Ltd., Kyoto, Japan)-based balance rehabilitation as an adjunct to conventional therapy, enhancing their dynamic and static balance, functional motor ability, and independence [17]. Marigold et al. suggested that muscle weakness and impairments in re-weighting and integrating information from the afferent cortex contribute to postural instability and falls in stroke patients [18]. Therapists can use this knowledge to devise effective interventions that improve postural control in these individuals.

In a study by Ghorbanpour et al., results indicated that anxiety increases internal focus on postural control and instability caused by stroke, leading to poor neuromuscular regulation of posture and heightened ankle muscle co-contraction, known as the ankle stiffening strategy, especially on unstable surfaces [19]. Among chronic stroke survivors with high anxiety levels, postural stability could be improved, and ineffective stiffening approaches minimized by redirecting attention from postural control using external focus or cognitive tasks [19]. Similarly, Peters et al. proposed that following a stroke, investigators and medical professionals can now monitor patients' activities in various contexts using smartphones and other wearable devices [20]. By leveraging wearable data, professionals can enhance their assessments and therapeutic plans by better understanding patients' real-world experiences [20].

Rito et al. suggested that VR balance training could significantly improve dynamic balance control compared to stationary balance training. These findings offer fundamental insights into recovering balance capacity following a stroke [21]. Jinlong et al. demonstrated that VR, which has a moderate to significant impact, enhances balance and motor function, particularly in the upper extremities, in stroke patients [22]. In a study by Cikajlo et al., patients who experienced a cerebrovascular accident required minimal assistance during rehabilitation sessions [23]. The study showed that various exercise games can target and improve balance, posture, one-leg standing, weight shifting, and muscle strength as effectively as conventional physical activity programs. The choice of exergames is essential when designing supplemental therapy for traditional rehabilitation regimens. Clinical evaluation using the 10-meter walk test, the Romberg test with closed eyes, and the four-square-step test revealed significantly positive outcomes [23]. VR gameplay can also help patients become more aware of their progress, ultimately contributing to a more effective stroke rehabilitation strategy enhancing their ability to perform daily activities.

Limitations of the study

Our study had several important limitations. We analyzed a small sample size and only included individuals within a specific age range, limiting the generalizability of our findings. The study duration was relatively short, which may not fully capture the long-term effects of the interventions. Additionally, the study design did not account for potential confounding variables such as participants' baseline physical activity levels, comorbidities, and adherence to the rehabilitation protocol outside the supervised sessions. The lack of a control group that received no VR or gait exercises also limits the ability to attribute improvements solely to the interventions studied.

Recommendations for future studies

Future studies should include larger and more diverse populations, and extend the duration of follow-up, futuristic VR modalities, and follow-up assessments could also provide robust results along with considering these additional variables to provide a more comprehensive understanding of the efficacy of VR-based rehabilitation protocols.

## Conclusions

This study demonstrated that immersive VR training combined with functional gait exercises improves dynamic balance and postural stability more than immersive VR training alone. These findings suggest that integrating VR with functional gait exercises into rehabilitation protocols can enhance recovery outcomes for stroke patients. This combined approach can lead to faster and more effective improvements in motor functions, increased independence, and a higher quality of life. The ability of VR to simulate real-life activities and provide immediate feedback allows for personalized rehabilitation programs tailored to each patient's specific needs. This customization can improve patient adherence and participation in rehabilitation programs. Further research with larger and more diverse populations is necessary to validate these findings and optimize the implementation of VR-based rehabilitation protocols.
